# Putting the Pieces Into Place: A Case of Systemic Polyarteritis Nodosa

**DOI:** 10.7759/cureus.8442

**Published:** 2020-06-04

**Authors:** David Brodell, Glynis Scott, Molly Plovanich

**Affiliations:** 1 Dermatology, University of Rochester Medical Center, Rochester, USA; 2 Dermatopathology, University of Rochester Medical Center, Rochester, USA

**Keywords:** polyarteritis nodosa, splinter hemorrhage

## Abstract

This is a case of systemic polyarteritis nodosa (PAN) in a 43-year-old male who initially presented to the hospital with a puzzling collection of signs and symptoms, including fever, arthralgias, myalgias, abdominal pain, dark urine, and rash. His illness evolved over the course of four weeks, and skin biopsy helped to clinch the diagnosis and lead to appropriate treatment. It is important to consider systemic PAN in the work-up of patients with subtle skin findings in the context of seemingly unrelated constitutional, abdominal, genitourinary, cardiac, and neurological signs and symptoms.

## Introduction

The systemic form of polyarteritis nodosa (PAN) is rare, often difficult to diagnose, and can be life threatening. In Europe, the annual estimated incidence is only two to nine per million people [1]. Per the Revised International Chapel Hill Consensus, the finding of necrotizing medium and small arteritis without glomerulonephritis or vasculitis in arterioles, capillaries, or venules, coupled with negative antineutrophilic cytoplasmic antibodies (ANCA), is consistent with a diagnosis of systemic PAN [2]. In addition, there are separate diagnostic criteria proposed by the American College of Rheumatology (ACR), including (1) weight loss >4 kilograms (kg); (2) livedo reticularis; (3) testicular pain or tenderness; (4) myalgias, weakness, or leg tenderness; (5) mononeuropathy or polyneuropathy; (6) diastolic blood pressure >90 mmHg; (7) elevated blood urea nitrogen (BUN) >40 mg/dL or creatinine >1.5 mg/dL not attributable to dehydration or obstruction; (8) presence of hepatitis B surface antigen or antibody in serum; (9) arteriogram showing aneurysms or occlusions of the visceral arteries not attributable to arteriosclerosis, fibromuscular dysplasia, or other non-inflammatory causes; and (10) biopsy of small or medium-sized artery containing polymorphonuclear neutrophils [3]. Per the ACR, meeting three out of the 10 criteria establishes a diagnosis of PAN with a sensitivity and specificity of 82.2% and 86.6%, respectively [3]. Regardless of the diagnostic criteria applied, it is important to have a high index of suspicion for this systemic vasculitis, as failure to initiate treatment can result in significant morbidity and mortality. Here, we present a case of systemic PAN in which recognition of skin changes involving the feet and nails led to definitive diagnosis.

## Case presentation

A 43-year-old man presented with six weeks of intermittent fever, arthralgias, myalgias, abdominal pain localized to the left lower quadrant, and rash. Prior to admission, he had been treated presumptively for diverticulitis with antibiotics with partial improvement in his abdominal pain. Shortly after initiation of antibiotics, he developed a rash, prompting a short course of steroids. This temporarily resulted in resolution of the rash and his constellation of symptoms. However, within a week of discontinuing steroids, he had recurrent fever and systemic symptoms, prompting admission.

Diagnostic evaluation on admission was notable for the following normal studies: serum creatinine, serum blood urea nitrogen, liver function panel, hematocrit, and platelets. His white blood cell count was elevated to 15,000/mL with 86% neutrophils, his C-reactive protein (CRP) was elevated to 256 mg/L, and his erythrocyte sedimentation rate (ESR) was elevated to 32 mm/hr. His urinalysis revealed 3+ blood with >50 red blood cells (RBCs) on microscopic analysis, but was negative for protein, nitrite, or leukocyte esterase. Blood cultures on admission demonstrated no growth.

**Figure 1:**
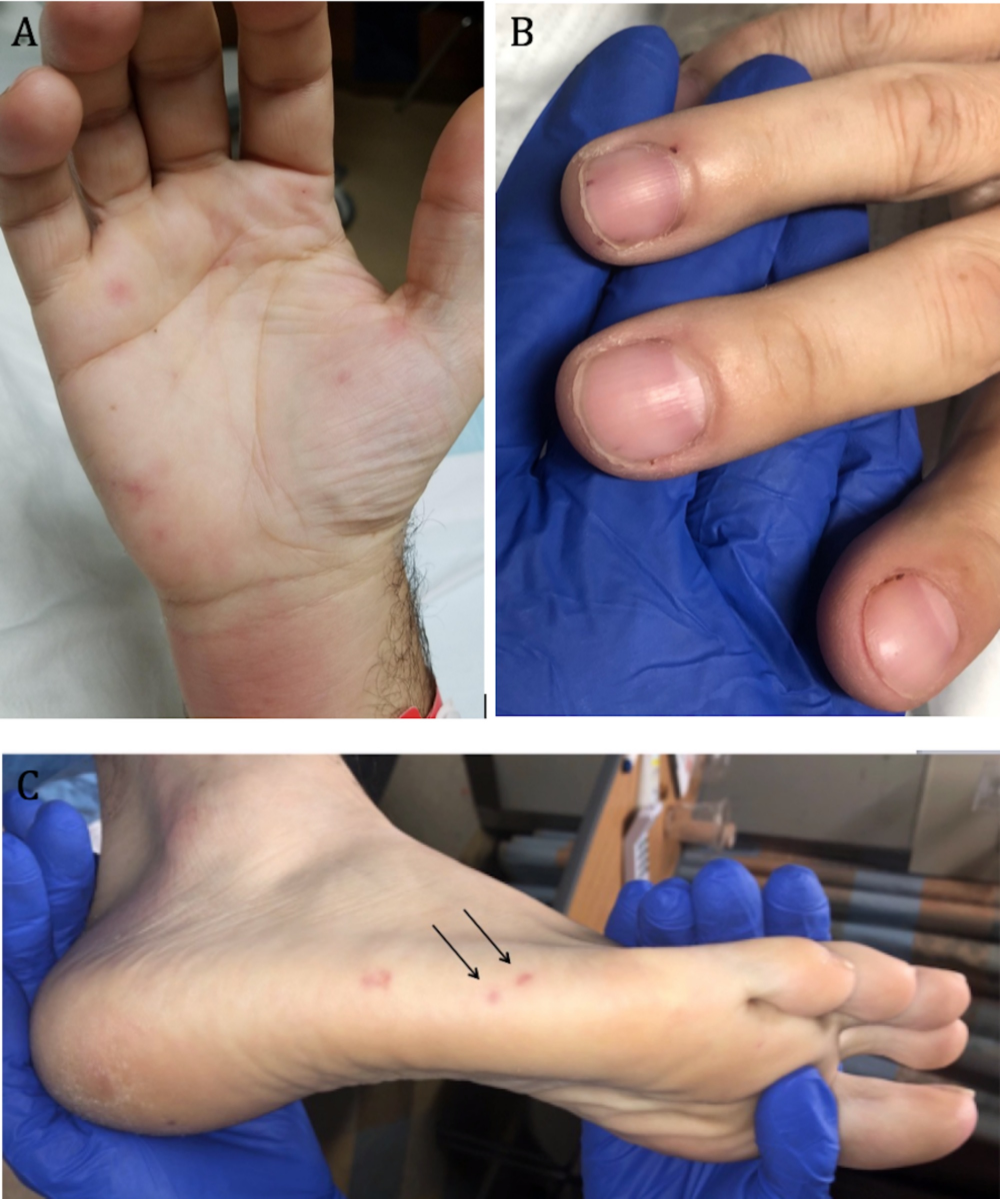
Cutaneous Manifestations in Our Patient With Systemic Polyarteritis Nodosa Representative cutaneous findings, including (A) tender palmar purpuric macules, (B) splinter hemorrhages, and (C) tender purpuric macules on the lateral aspect of the right foot.

On exam, he had a rash consisting of scattered pink papules on the arm, legs, and forehead, as well as a few purpuric macules on the palmoplantar surfaces, prompting biopsy of a papule on the right arm. This showed a sparse dermal lymphocytic and neutrophilic infiltrate, possibly consistent with a neutrophilic dermatosis, such as Sweet syndrome. He was treated with prednisone 1 mg/kg for three days for a presumptive diagnosis of Sweet syndrome with minimal improvement. Although the rash on his body improved, his fevers, systemic symptoms, and the purpuric macules on his palmoplantar surfaces persisted (Figure 1A), the latter of which become increasingly tender. Additionally, he was noted to have new splinter hemorrhages involving three fingernails (Figure 1B). At this point, the differential included infectious endocarditis, disseminated gonococcal infection, an embolic process (perhaps, due to a cardiac mass), a thrombotic disorder (such as anti-phospholipid syndrome), and systemic vasculitis. He then abruptly developed chest pain and was diagnosed with an ST elevation myocardial infarction requiring intervention to an occluded circumflex artery. To assist with diagnosis, he underwent repeat skin biopsy from a purpuric macule on his lateral foot (Figure 1C).

**Figure 2:**
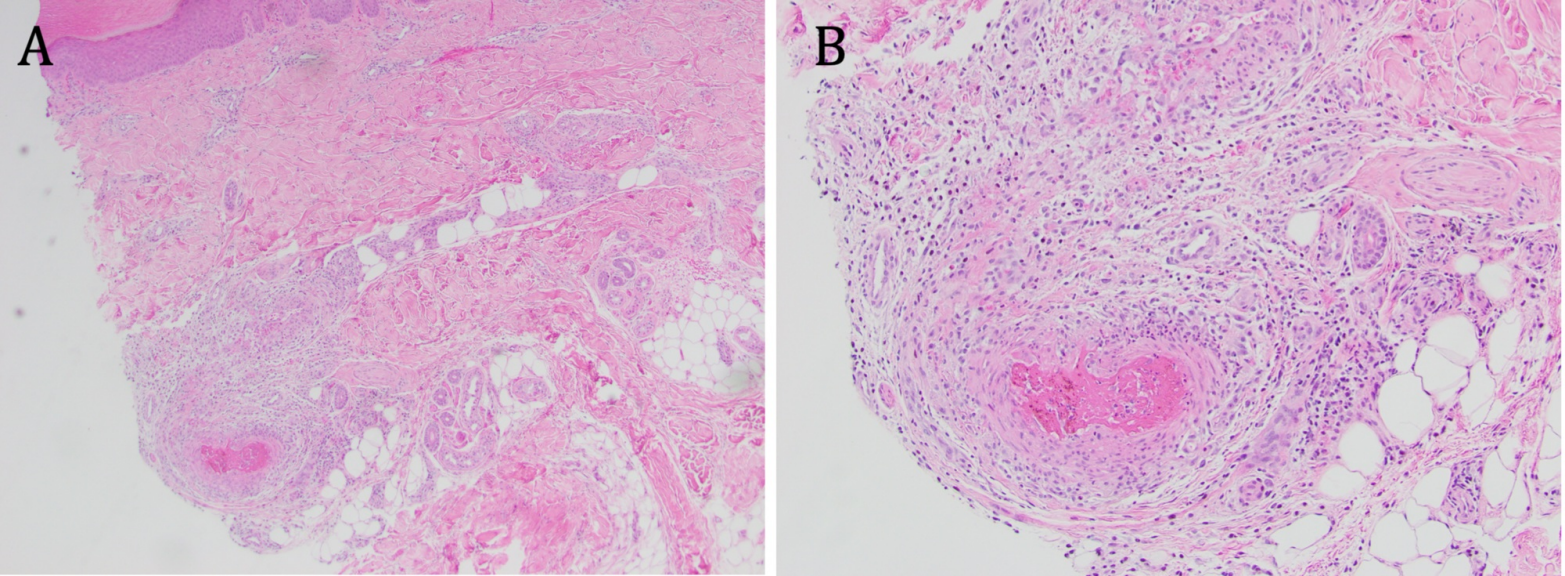
Biopsy From Right Lateral Foot Demonstrating Histological Findings of Systemic Polyarteritis Nodosa Here we see a medium caliber artery with intravascular thrombi and focal vasculitis without involvement of small caliber vessels or capillaries, overall consistent with polyarteritis nodosa, including at (A) x4 and (B) x10.

This demonstrated a medium caliber artery with intravascular thrombi and focal vasculitis without involvement of small caliber vessels or capillaries, overall consistent with PAN (Figure 2). Stains for organisms, including Brown and Brenn, acid-fast bacilli (AFB), and Grocott methenamine silver (GMS), were negative. Tissue cultures obtained from a purpuric macule demonstrated no growth on aerobic, AFB, and fungal media. He underwent transthoracic and transesophageal echocardiogram, which did not reveal signs of either a cardiac mass or vegetation. Several sets of blood cultures demonstrated no growth. Testing for hepatitis B, hepatitis C, HIV, EBV, and parvovirus was negative. Taken together, these findings were consistent with a diagnosis of systemic PAN.

He began treatment with high-dose steroids (IV methylprednisolone 1 g daily) and IV cyclophosphamide. On the day he was scheduled to start cyclophosphamide, he developed progressive headache and visual changes, prompting MRI which demonstrated multiple punctate acute and subacute infarcts involving the bilateral cerebral hemispheres and foci of microhemorrhage involving the cerebral hemispheres, the cerebellar hemispheres, and the brain stem; additionally, there was enhancement of the distal arteries, consistent with central nervous system (CNS) vasculitis. Subsequently, he underwent diagnostic cerebral angiography, which showed an aneurysm of the right posterior communicating artery, as well as signs of CNS vasculitis elsewhere, including dilation, irregularity, and branch blocks within branches of the right internal carotid artery. His hospital course was complicated by Clostridium difficile colitis and Rothia mucilaginosa bacteremia, which were successfully treated with antibiotics. Over the course of the next month, he slowly responded to cyclophosphamide and was discharged home.

## Discussion

This was a challenging case of a middle-aged man presenting with a constellation of systemic findings, ultimately diagnosed as systemic PAN. Multiple alternative diagnoses were entertained, including inflammatory bowel disease (early in his course given his abdominal pain), Sweet syndrome (after his first skin biopsy), infectious endocarditis, and underlying thrombophilia. In his case, recognition of cutaneous findings, including tender purpuric macules on the palmoplantar surfaces and splinter hemorrhages, and skin biopsy, was pivotal in reaching the correct diagnosis. Interestingly, a second biopsy was required to establish the unifying diagnosis of systemic PAN. The finding of a neutrophilic dermatosis instead of PAN in the first biopsy may be attributed to a sampling error as we may not have captured an involved vessel on our first punch biopsy. Alternatively, he may have developed Sweet syndrome as a reactive phenomenon to his systemic vasculitis.

Although skin findings are less commonly reported in association with systemic PAN relative to cutaneous PAN, between 28% and 60% of patients with systemic PAN have palpable purpura (most commonly), livedo reticularis (as reflected in the ACR diagnostic criteria), nodules, urticaria, or less commonly, digital necrosis and splinter hemorrhages [4,5]. Our patient demonstrated urticarial-like lesions, tender purpuric macules, and splinter hemorrhages. Ultimately, skin biopsy of a tender purpuric macule demonstrated a segmental necrotizing vasculitis, clinching the diagnosis of systemic PAN. 

This case also highlights the importance of revisiting a diagnosis as a patient’s clinical picture evolves. Early in his course, his constellation of symptoms and skin biopsy appeared to fit with Sweet syndrome. However, given the failure to respond to prednisone and the development of new clinical findings (including myocardial infarction and CNS involvement), we revisited the working diagnosis and obtained additional diagnostic information in collaboration with a multidisciplinary team, including internal medicine, rheumatology, infectious disease, neurology, and cardiology, ultimately reaching the correct diagnosis. Reflecting on the ACR diagnostic criteria for systemic PAN, our patient met five out of 10 criteria, including weight loss, myalgias, diastolic blood pressure >90 mmHg, arteriogram with aneurysms, and biopsy demonstrating characteristic vasculitis. Of note, this patient’s hepatitis B testing was negative. Previously, prior to hepatitis B vaccination, more than one-third of patients had hepatitis B-associated PAN, but at present, it is estimated that only ~5% of cases are associated with hepatitis B [4]. Other important disease associations to consider include hepatitis C and hairy cell leukemia.

Treatment of systemic PAN involves prompt diagnosis, systemic steroids (often starting with IV methylprednisolone at a dose of 15 mg/kg daily with a maximum of 1 g daily), and cyclophosphamide, either IV or oral [6]. Our patient was treated with IV methylprednisolone 1 g for three days, which was then converted to oral prednisone 1 mg/kg daily, and IV cyclophosphamide 600 mg/m^2^ for two doses, after which it was increased to 750 mg/m^2^ given new cerebral aneurysm formation. He continues on monthly cyclophosphamide infusions at 750 mg/m^2^, which he will continue for six months while tapering his prednisone.

The reported mortality associated with systemic PAN is 24.6%, highlighting the life-threatening nature of this systemic vasculitis [7]. Renal failure and organ infarction, involving the bowel, heart, and brain, are the primary causes of death. The five-factor score, derived from the French Vasculitis Study Group, risk stratifies patients, providing an estimate of five-year mortality. For systemic PAN, there are four clinical factors that portend a poor prognosis, including (1) age >65 years, (2) cardiac symptoms, (3) gastrointestinal involvement, and (4) renal insufficiency (plasma creatinine >1.7 mg/dL) [7].

## Conclusions

Systemic PAN, a small and medium arteritis, is a potentially life-threatening disease that can affect almost any system in the body. It may have constitutional, abdominal, genitourinary, cardiac, and neurological manifestations in the setting of tender purpuric acral lesions and splinter hemorrhages. Our patient’s subacute evolution highlights the need for a thoughtful approach and interdisciplinary care in reaching the proper diagnosis. Appropriate treatment mitigates significant morbidity and mortality.
